# Relations between BOLD fMRI-Derived Resting Brain Activity and Cerebral Blood Flow

**DOI:** 10.1371/journal.pone.0044556

**Published:** 2012-09-21

**Authors:** Zhengjun Li, Yisheng Zhu, Anna Rose Childress, John A. Detre, Ze Wang

**Affiliations:** 1 Department of Biomedical Engineering, Shanghai Jiao Tong University, Shanghai, People's Republic of China; 2 Department of Psychiatry, Perelman School of Medicine, University of Pennsylvania, Philadelphia, Pennsylvania, United States of America; 3 Department of Neurology, Perelman School of Medicine, University of Pennsylvania, Philadelphia, Pennsylvania, United States of America; University Of Cambridge, United Kingdom

## Abstract

Consistent resting brain activity patterns have been repeatedly demonstrated using measures derived from resting BOLD fMRI data. While those metrics are presumed to reflect underlying spontaneous brain activity (SBA), it is challenging to prove that association because resting BOLD fMRI metrics are purely model-free and scale-free variables. Cerebral blood flow (CBF) is typically closely coupled to brain metabolism and is used as a surrogate marker for quantifying regional brain function, including resting function. Assessing the correlations between resting BOLD fMRI measures and CBF correlation should provide a means of linking of those measures to the underlying SBA, and a means to quantify those scale-free measures. The purpose of this paper was to examine the CBF correlations of 3 widely used neuroimaging-based SBA measures, including seed-region based functional connectivity (FC), regional homogeneity (ReHo), and amplitude of low frequency fluctuation (ALFF). Test-retest data were acquired to check the stability of potential correlations across time. Reproducible posterior cingulate cortex (PCC) FC vs regional CBF correlations were found in much of the default mode network and visual cortex. Dorsal anterior cingulate cortex (ACC) FC vs CBF correlations were consistently found in bilateral prefrontal cortex. Both ReHo and ALFF were found to be reliably correlated with CBF in most of brain cortex. None of the assessed SBA measures was correlated with whole brain mean CBF. These findings suggest that resting BOLD fMRI-derived measures are coupled with regional CBF and are therefore linked to regional SBA.

## Introduction

The human brain consumes about 20% of the body's energy [1] and most of the “energy budget” in the brain is spent on the intrinsic or spontaneous activity supporting communication among neurons and their supporting cells [2]. This striking neurophysiologic phenomenon motivates the increasing interest in examining spontaneous brain activity (SBA) using BOLD fMRI data acquired at rest. Various data-derived resting brain activity measures have been assumed to reflect underlying SBA including seed-region based functional connectivity (SRFC), regional coherence, and regional low frequency fluctuations [2]–[12]. Evidence demonstrates that such SBA measures, derived from resting functional neuroimaging, can be altered by either functional tasks [8], [11], [13], [14] or brain disorders [15]–[18]. While these investigations support the notion of using resting BOLD fMRI-based SBA measures as potential biomarkers for brain disorders or treatment effects, most of these measures have yet to be linked with underlying brain metabolism. Examining that link is important for verifying the physiological significance of these SBA metrics since the metrics themselves are unitless.

Cerebral blood flow (CBF) is closely coupled with brain metabolism [19] and has been used an indirect measure of brain energy demand for over a century [20], [21]. Using PET imaging, changes in regional CBF have been shown to be comparable to changes in regional glucose metabolism in response to task activation [22]–[29]. Although this coupling is still in debate when the brain is under the condition of task activation [20], [21], [30], [31], it sustains since it was demonstrated in the resting brain more than two decades ago [11], [22], [23]. More evidence of the CBF-brain function coupling comes from arterial spin labeling (ASL) perfusion MRI, which provides a noninvasive methodology for quantifying CBF [32], [33] and has been increasingly adopted to visualize brain functions via assessing the resting CBF or its changes in response to exogenous stimuli or even medication [33]–[40]. In summary, CBF has been widely demonstrated to be coupled with brain functions and it has been shown to be coupled to brain metabolism, including during resting states. It is then reasonable to hypothesize that CBF is reflective of SBA and assessing the correlations between ASL CBF and resting BOLD fMRI metrics should provide a means of linking BOLD fMRI-based SBA metrics to the underlying brain energy demands.

Using ASL MRI, our group have demonstrated that brain regions within the default mode network have higher resting CBF than other places [41], which was replicated by Zou et al. [42]. Such similarity between resting CBF distribution and fMRI-derived SBA patterns suggests a possible correlation between the apparent SBA patterns and CBF. However, a direct SBA-CBF association study is still missing.

In this study, we acquired repeat resting ASL data and resting BOLD data from the same cohort of normal subjects to assess the potential SBA-CBF correlations and their test-retest stability. Part of this work has been presented in a conference [43] (see the File S1).

## Materials and Methods

### Subjects

The resting BOLD fMRI data have been used in a recent publication [44]. 15 young healthy subjects (mean age = 25, range = 20–35, SD = 4.75, 8 male) were scanned twice 2 months apart. Signed written consent forms approved by local IRB were collected before each of the scan. Careful screening was conducted to exclude any neurological diseases that could alter brain blood flow or brain functions. The subjects had no history of drug or alcohol abuse and were not using any medication that can alter blood flow. They did not make any long-distance travel during the 2 months before the second MR scan so they had no exposure to jet lags which might change blood flow. All subjects were told to not drink any caffeinated beverages 12 hours before the scan time.

### Image acquisition

MR imaging was conducted in a 3-T whole-body scanner (Siemens Medical Systems, Erlangen, Germany). Each session contained an anatomical scan, a resting ASL scan, and a resting BOLD fMRI scan. High-resolution structural images were acquired for spatial brain normalization using a 3D MPRAGE sequence (TR/TE/TI = 1620/3/950 ms). ASL imaging utilized an amplitude modulated continuous ASL (CASL) perfusion imaging sequence optimized for 3.0 T [45] with a standard transmit/receive (Tx/Rx) head coil (Bruker BioSpin, USA). The head coil and foam pads were positioned carefully to reduce head movement. Acquisition parameters were TR = 3.8 s, TE = 17 ms, FOV = 220×220 mm^2^, matrix = 64×64×12, slice thickness = 7 mm, inter-slice space = 2.35 mm, labeling time = 2 s, post label delay time = 1 sec, bandwidth = 3 kHz/pixel, flip angle = 90°. 50 label/control image pairs were acquired for each subject. Gradient-echo echo-planar imaging sequence was used for BOLD fMRI data acquisition with parameters of: TR = 3 s, TE = 30 ms, FOV = 220×220 mm^2^, matrix = 64×64, 40 interleaved slices with thickness = 3 mm, 220 images. Participants were asked to lie still in the scanner at rest, keep their eyes open, and think about nothing.

### Data analysis

The following data processing was used for both sessions of BOLD data and ASL data.

### Image preprocessing

All BOLD resting data preprocessing was performed using SPM8 (http://www.fil.ion.ucl.ac.uk/spm) based batch scripts [46]. The processing steps consisted of: realignment, coregistration, smoothing with an isotropic Gaussian filter (FWHM = 6 mm), low-pass filtering with a Butterworth filter (cutoff frequency = 0.08 Hz), and a high-pass Butterworth filtering (cutoff frequency = 0.009 Hz). Diffeomorphic Anatomical Registration Through Exponential Lie Algebra (DARTEL) [47] was used to generate a local template for all subjects based on their high-resolution structural images and warp each individual's BOLD images to the local template space. For each individual subject, a brain mask was generated based on the mean control image after skull stripping. This mask was used to calculate the whole brain mean signal for each acquired BOLD image. The time course of that mean signal was termed the global BOLD signal. The CSF and white matter ROIs were defined as spheres of 6 mm radius within CSF and white matter, and were used to extract mean CSF signal and WM signal as nuisances to be included in SPM8. Head motion time courses, the global BOLD signal time course, the mean CSF signal time course and mean white matter signal time course were filtered out from the normalized BOLD images at each voxel using simple regression [48].

ASL images were motion corrected using the same routine as that used for BOLD data after removing the effects of spin labeling by regressing out the labeling paradigm [49]. No low-pass filtering was applied for ASL data. Other preprocessing steps were the same as those for BOLD images and have been described in the ASL data processing pipeline implemented in the ASL data processing toolbox, ASLtbx [46]. One mean CBF map was generated from the 50 label/control ASL image pairs. The above mentioned whole brain mask was used to calculate each subject's whole brain mean CBF, which is called global CBF hereafter. The mean CBF images were normalized to the local template space using the same transformation matrix generated by DARTEL.

### Seed region-based functional connectivity

Seed regions of interest (ROI, voxels within a sphere of 6 mm radius) were defined in the anterior cingulate cortex (ACC) and posterior cingulate cortex (PCC) using the Pickatlas utility [50]. Because ACC is relatively a large brain region with different functions in different sections [51], 4 ACC ROIs were used including 3 in the ventral ACC (vACC) and 1 in the dorsal ACC (dACC). The mean signals of the ACC ROIs and PCC ROI were extracted from each subject's spatially normalized BOLD resting images and were subsequently used as regressors in a whole brain linear regression analysis, respectively. For each subject, all voxels' correlation coefficients of the two SRFC analyses were collected as the ACC-FC and PCC-FC maps, respectively.

### Regional coherence

The Kendall's coefficient concordance (KCC, also known as Kendall's W) [52], and called ReHo in [53], was calculated at each voxel using a predefined neighborhood. A sphere with a radius of 6 mm was used in this paper. Kendall's W ranges from 0 to 1, where 0 means no coherence and 1 means coherent. The collection of all voxels' Kendall's W formed the so-called ReHo map.

### ALFF

Each voxel's BOLD time series was transformed into the frequency domain using MATLAB (Mathworks Inc., Natick, MA) and the mean amplitude of the spectrum over the frequency range of 0.01–0.08 Hz was calculated as the ALFF [54].

### Group analysis

Across-subjects average CBF, ReHo and ALFF maps were calculated to illustrate the spatial distribution patterns of each measure. Each subject's CBF, ReHo and ALFF images were divided by the whole brain mean of each type of maps to generate relative maps of each metric. For each of the 3 relative maps (CBF, ReHo, and ALFF), a one sample t-test was performed at each voxel to assess whether the across subject mean is significantly different from 1 or not. The positive t-map and negative t-map were used to identify brain regions with significantly higher (or lower) than average CBF/ReHo/ALFF values, respectively, for each type of the 3 measures. The same analyses were performed for each scan session (day 1 and day 2) separately.

#### SBA-CBF association analyses

Pearson's correlation coefficient (CC) calculated in MATLAB was used to assess correlations between 1) global SBA vs global CBF, 2) regional SBA at each voxel vs global CBF, and 3) regional SBA vs regional CBF. In analysis 1, CC was directly calculated from the global SBA values and the global CBF values of all subjects. Global SBA was calculated from the corresponding SBA maps using the same brain mask used for calculating whole brain mean CBF. In analysis 2, CC at each voxel was calculated between the global CBF values and SBA values of that voxel from all subjects. CC's of all voxels were grouped into a CC map. In analysis 3, CC at each voxel was calculated between that voxel's CBF values and SBA values from the same subjects. CC's of all voxels were grouped into a CC map. The same association analyses were performed for both of 2 scan sessions. ReHo vs CBF ratio and ALFF vs CBF ratio at each voxel was calculated to directly demonstrate the spatial homogeneity of the ReHo-CBF and ALFF-CBF correlations.

The CBF/FC/ReHo/ALFF distribution analysis results were thresholded at p<0.001 and the voxel-wise SBA vs CBF correlation results were thresholded at p<0.005 (CC>0.683, uncorrected for multiple comparisons). The results were also thresholded with cluster extent >30 voxels. The overlap percentage of the two scan sessions' suprathreshold SBA vs CBF CC maps was calculated to evaluate the test-retest reliability for each of the assessed SBA-CBF associations.

An additional analysis was performed to test whether various brain networks can be ranked or quantified using their CBF values. Brain networks were identified using group independent component analysis (ICA) network analysis [55] on all subjects' BOLD images (after being registered to the MNI space) using MELODIC (Multivariate Exploratory Linear Decomposition into Independent Components) Version 3.09, part of FSL (FMRIB's Software Library, www.fmrib.ox.ac.uk/fsl). Two resting state networks were identified as examples to test the hypothesis: the default mode network (DMN) and executive control network (ECN) [4]. The mean CBF in each of the two network regions were extracted for each subject and statistically compared for each session.

## Results

### Group level distributions of CBF, ReHo and ALFF

**Figure 1:**
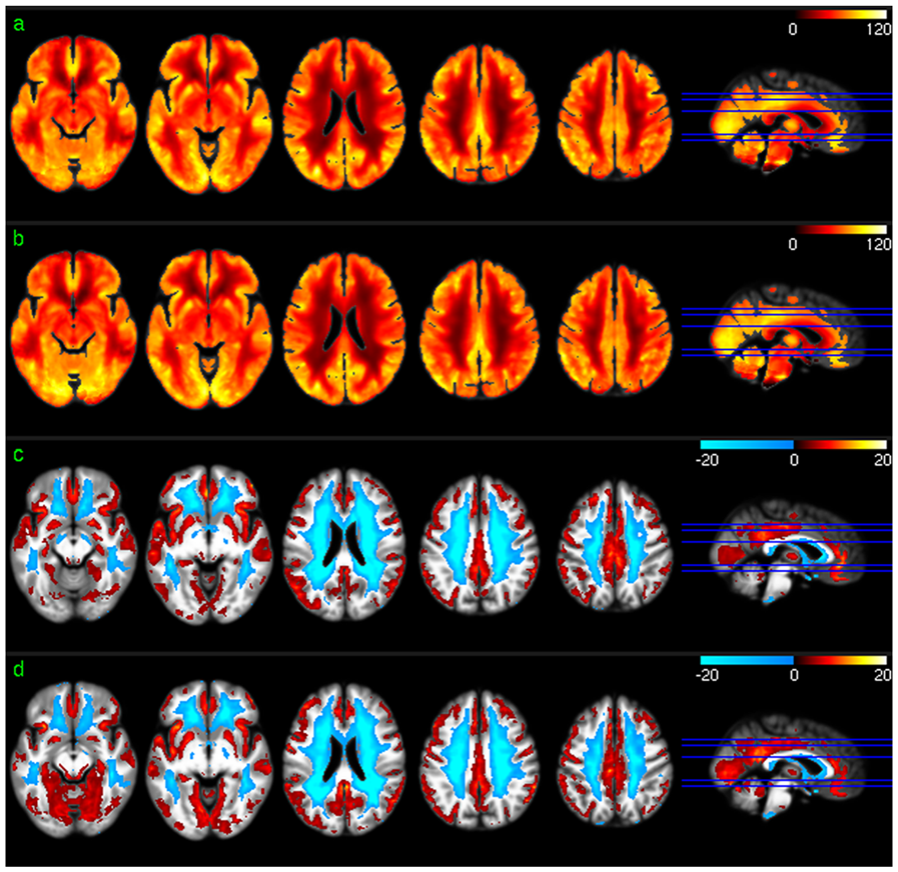
Group level CBF analysis results. a) Mean CBF map of session 1, b) mean CBF map of session 2, c) CBF distribution map of session 1, and d) CBF distribution map of session 2. Red and blue in c) and d) mean higher than average CBF and lower than average CBF, respectively. c) and d) were thresholded with p<0.001 (uncorrected).

Fig. 1 shows the across-subject resting mean CBF maps (Fig. 1a and 1b) and the group level resting CBF distribution patterns (Fig. 1c and 1d) for scan session 1 and session 2, respectively. As shown in the results of group level one sample T test based on the relative CBF images (Fig. 1c and 1d), higher than average CBF was found in medial orbitofrontal cortex (mOFC), frontal cortex (FC), cingulate cortex, insula, middle and superior temporal cortex (TC), putamen, precuneus, bilateral parietal cortex (PC), and visual cortex (VC) in both sessions. White matter showed lower than average CBF, as expected.

**Figure 2:**
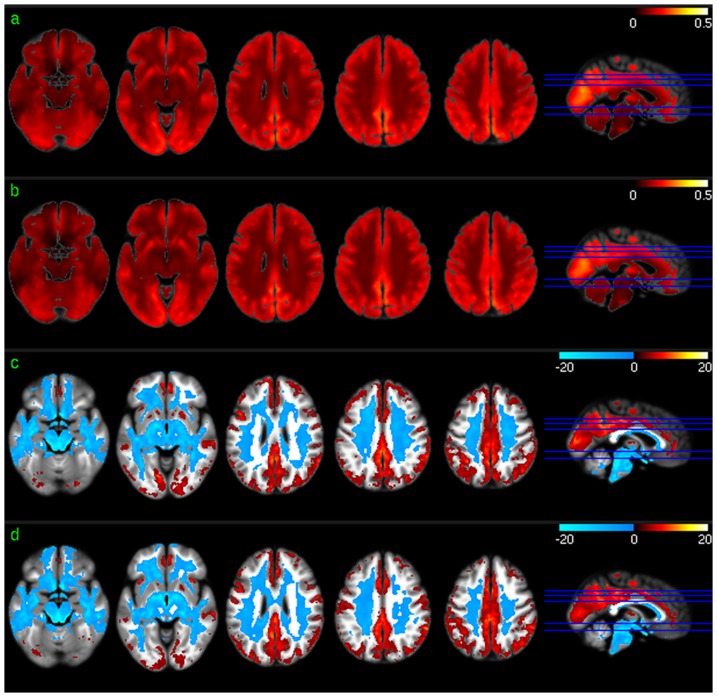
Group level ReHo analysis results. a) Mean ReHo map of session 1, b) mean ReHo map of session 2, c) ReHo distribution map of session 1, and d) ReHo distribution map of session 2. Red and blue in c) and d) mean higher than average ReHo and lower than average ReHo, respectively. c) and d) were thresholded with p<0.001 (uncorrected).

Fig. 2 shows the group level results of ReHo analysis. Except for a scale difference, the mean ReHo maps (Fig. 2a and 2b) appeared to be very similar to the CBF maps as shown in Fig. 1a and 1b. ReHo in WM were found to be more homogeneous than CBF in WM. ReHo distribution patterns (Fig. 2c and 2d) were similar to CBF distribution patterns (Fig. 1c and 1d) in most brain areas. The lower than average ReHo pattern in inferior thalamus, temporal lobe, and inferior insula (the left 2 slices in Fig. 2c, 2d) was more spatially distributed than the lower than average CBF pattern (the left 2 slices in Fig. 1c, 1d), while the higher than average ReHo pattern in VC and inferior TC (iTC) (the left 2 slices in Fig. 2c, 2d) was less spatially distributed than that of the higher than average CBF pattern (the left 2 slices in Fig. 1c, 1d).

**Figure 3:**
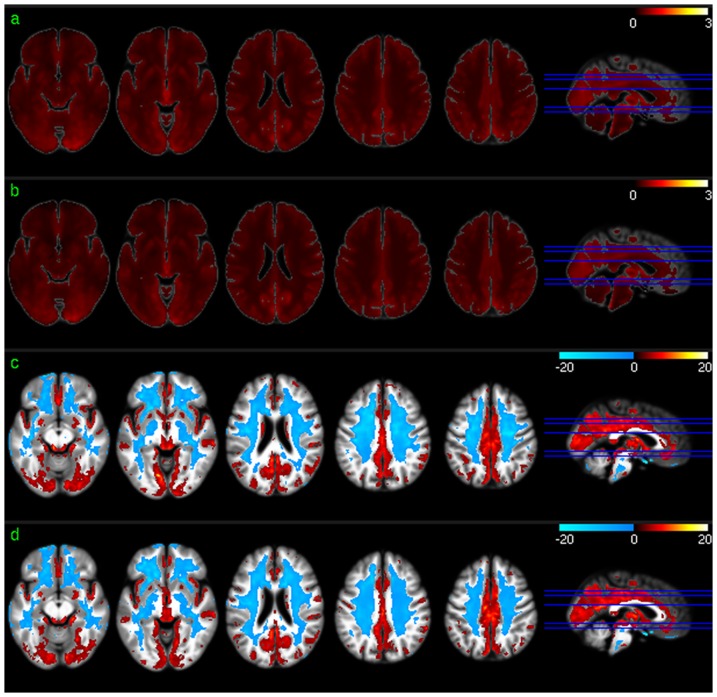
Group level ALFF analysis results. a) Mean ALFF map of session 1, b) mean ALFF map of session 2, c) ALFF distribution map of session 1, and d) ALFF distribution map of session 2. Red and blue in c) and d) mean higher than average ALFF and lower than average ALFF, respectively. c) and d) were thresholded with p<0.001 (uncorrected).

Fig. 3 shows the group level results of ALFF analysis. The mean ALFF maps (Fig. 3a, 3b) demonstrated similar GM/WM contrast to that of the mean CBF maps (Fig. 1a, 1b) or the mean ReHo maps (Fig. 2a, 2b). Significant higher than average ALFF was found in cingulate cortex, precuneus, bilateral PC, VC, putamen, insula, fusiform, and middle TC (mTC). Similar to the group level results of CBF and ReHo analysis, ALFF in WM was significantly lower than average.

### Group level PCC-FC and ACC-FC

**Figure 4:**
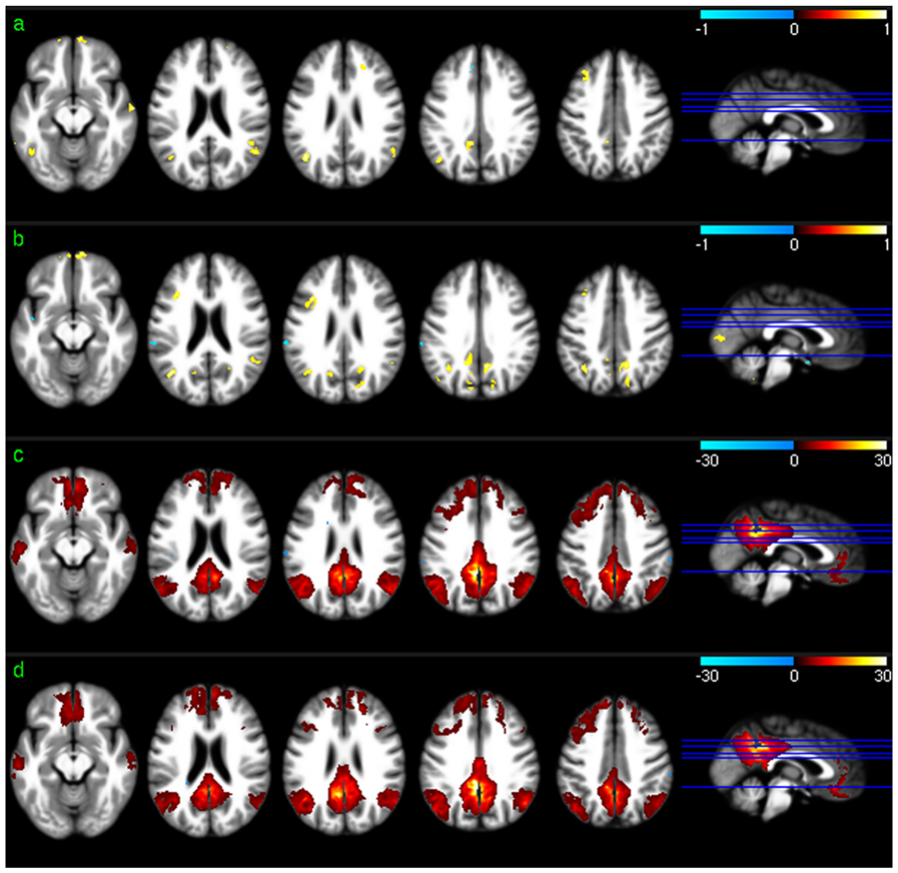
PCC-FC vs regional CBF association analysis results. a) and b) are the PCC-FC vs CBF correlation map (thresholded with p<0.005, uncorrected) for session 1 and 2, respectively. c) and d) are the group level PCC-FC maps (p<0.001, uncorrected) for session 1 and 2, respectively.

**Figure 5:**
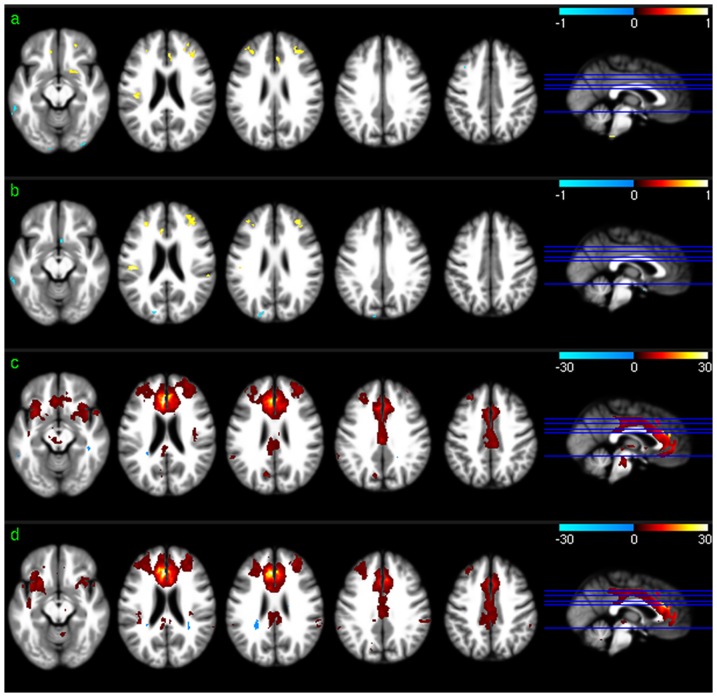
dACC-FC vs regional CBF association analysis results. a) and b) are the dACC-FC vs CBF correlation map (thresholded with p<0.005, uncorrected) for session 1 and 2, respectively. c) and d) are the group level dACC-FC maps (p<0.001, uncorrected) for session 1 and 2, respectively.

Significant PCC-FC correlations were found in PCC, ACC, mOFC, prefrontal cortex (PFC), mTC, bilateral PC, and precuneus in both sessions (Fig. 4c and 4d). Significant dACC-FC (Fig. 5c and 5d) correlations were found in ACC including vACC and dACC, mOFC, insula, and bilateral PFC. (vACC-FC maps were not displayed because no significant correlations were found between regional CBF and vACC-FC using any of the 3 vACC ROIs).

### SBA-CBF association analyses results

No significant correlations were found between global CBF and any of the 4 SBA metrics either globally (global CBF vs global SBA) or regionally (global CBF vs each voxel's SBA). Both global ReHo and global ALFF showed a trend of correlation to global CBF. The CC of global ReHo vs global CBF was 0.19 and 0.28 for session 1 and session 2, respectively; CC of global ALFF vs global CBF was 0.41 and 0.43 for session 1 and session 2, respectively.

Significant voxel-wise correlation between regional absolute CBF and PCC-FC in mOFC, VC, PCC/precuneus, bilateral PC, and left dorsal lateral PFC (DLPFC) were demonstrated in both sessions (Fig. 4a and 4b). Most of these positively correlated regions are in the significant PCC FC regions shown in Fig. 4c and 4d. Correlations in other brain regions were not reproduced in both test-retest sessions. 11.68% of the suprathreshold voxels of the two sessions overlapped.

**Figure 6:**
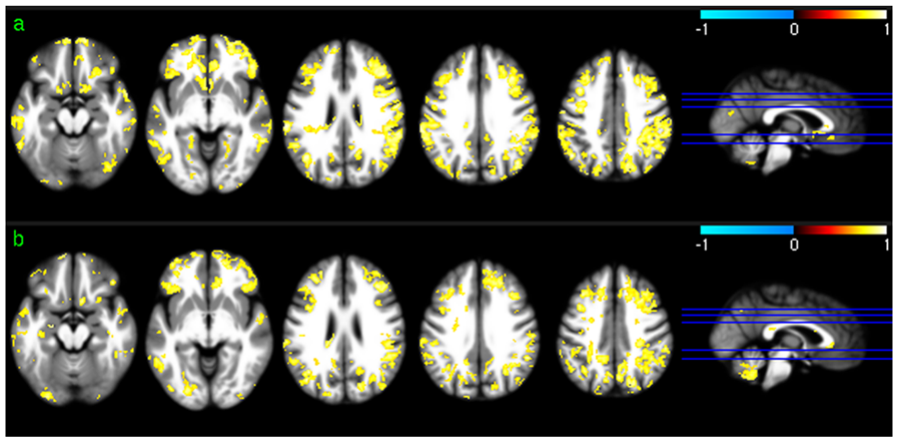
ReHo vs regional CBF correlation maps for a) session 1 and b) session 2 thresholded at p<0.005 (uncorrected).

**Figure 7:**
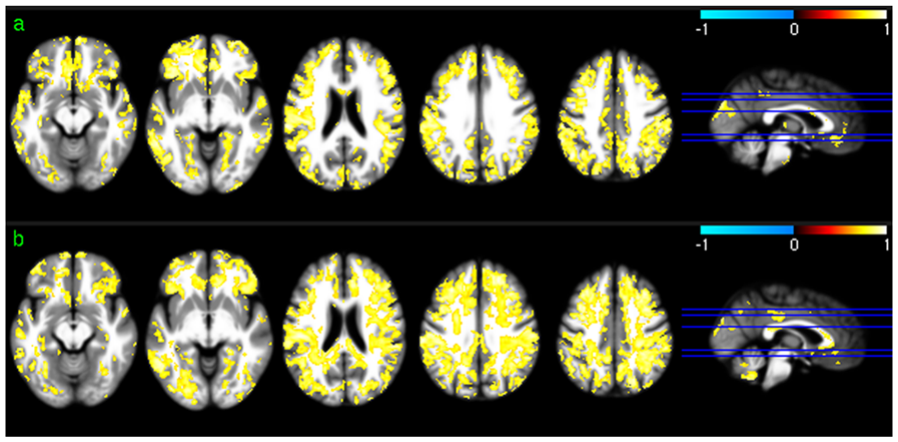
ALFF vs regional CBF correlation maps for a) session 1 and b) session 2 thresholded at p<0.005 (uncorrected).

Positive voxel-wise correlations between regional CBF and dACC-FC were consistently demonstrated in bilateral PFC in both sessions (Fig. 5a and 5b), which overlapped with the significant dACC-FC regions as shown in Fig. 5c and 5d. Repeat CBF vs dACC-FC correlations in iTC did not overlap with the significant dACC-FC correlations. CBF vs dACC-FC correlations in other areas were not reproducible. 10.72% of the suprathreshold voxels of the two sessions overlapped. Reproducible regional CBF vs ReHo correlations were found in bilateral TC, mOFC, lateral orbitofrontal cortex (lOFC), bilateral DLPFC, bilateral PC, and precuneus in both the test and retest sessions (Fig. 6a and 6b). 33.59% of the suprathreshold voxels of the two sessions overlapped. ALFF was found to correlate with CBF across both test-retest sessions in most of the brain cortices except for middle and posterior insula, putamen, PCC, and left iTC (Fig. 7). 44.42% of the suprathreshold voxels of the two sessions overlapped.

**Figure 8:**
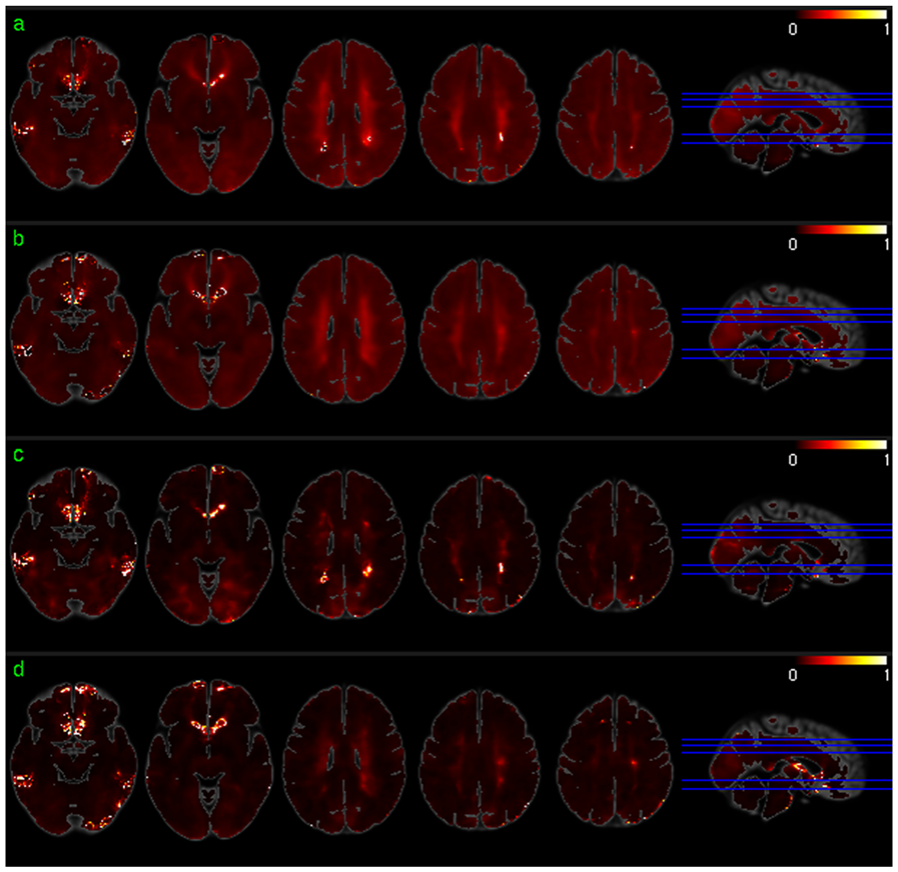
The mean and standard deviation maps of the ReHo/CBF ratio. a) and b) are the mean ReHo/CBF ratio map for session 1 and session 2, respectively. c) and d) are the standard deviation maps of the ReHo/CBF ratio for session 1 and 2, respectively. The map intensity was multiplied by 10000 for the purpose of illustration.

**Figure 9:**
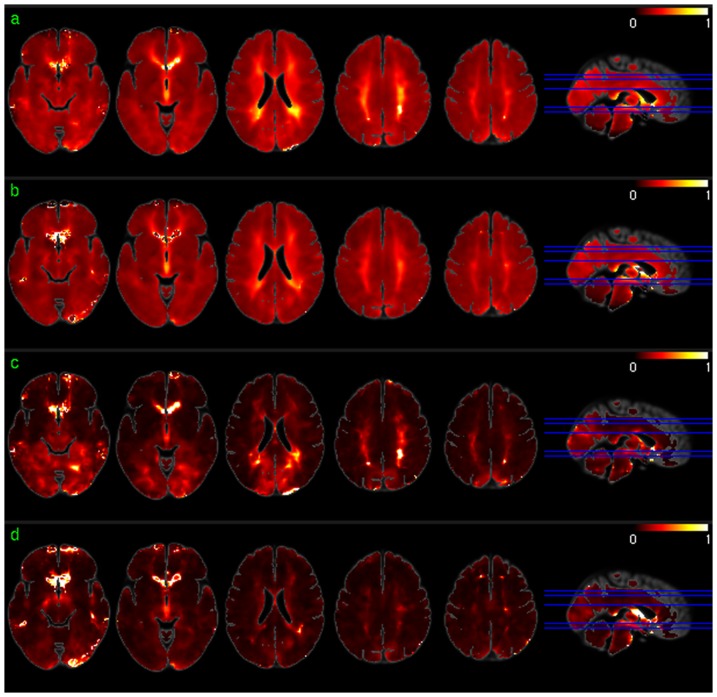
The mean and standard deviation maps of the ALFF/CBF ratio. a) and b) are the mean ALFF/CBF ratio maps for session 1 and session 2, respectively. c) and d) are the standard deviation maps of the ALFF/CBF ratio for session 1 and 2, respectively. The map intensity was multiplied by 10000 for the purpose of illustration.

Fig. 8 and 9 show the mean and standard deviation maps of the ReHo/CBF (Fig. 8) and ALFF/CBF (Fig. 9) ratios for both scan sessions. A uniform and stable (low standard deviation) ReHo/CBF ratio was found throughout GM except for part of iTC, precuneus, and VC in both sessions (Fig. 8). WM and ventricle areas showed high ReHo/CBF ratios with high standard deviations. Similar findings occurred in the ALFF/CBF ratio analysis (Fig. 9).

## Discussion

Regional CBF obtained with ASL MRI provides a means to assess the links between resting BOLD imaging-derived SBA measures and underlying brain metabolism. This study provides the first evidence that the resting BOLD imaging-based SBA measures are related to regional CBF.

Group level CBF, FC, ReHo, and ALFF patterns were first assessed for each of the 2 scan sessions to check the similarity of the distribution patterns of CBF and each of the 3 SBA measures (FC, ReHo, and ALFF). Consistent with the findings reported in the literature [11], [41], [42], a set of reliable high CBF (higher than whole brain mean) regions was found in both sessions, consisting of mOFC, lOFC, PFC, putamen, insula, temporal cortex, ACC, PCC, VC, precuneus, and bilateral PC. The significant high ReHo regions overlapped with the high CBF regions in mOFC, PFC, ACC, putamen, PCC, precuneus, and bilateral PC, suggesting that the relatively high coherent resting brain activity in those regions is associated with increased resting brain metabolism. A set of high ALFF (higher than whole brain average) regions was identified in mOFC, insula, iTC, putamen, VC, PCC, precuneus, VC, and bilateral PC, which also overlaps with the high CBF and high ReHo regions described above. This overlap suggests that increased brain metabolism might be required to support regionally coherent and slowly fluctuating resting brain activity. WM showed lower than average CBF/ReHo/ALFF as expected, and WM CBF is known to be lower than GM CBF [56]. Low WM ReHo and WM ALFF suggest that resting brain activity in WM is sporadic across voxels and random across time, which may require relatively lower energy support than that for GM, as reflected by the relatively lower CBF. The overlap between the high CBF regions and PCC-FC and dACC-FC network regions suggest a potential CBF modulation for each of the FC in the overlapped regions.

Repeatable regional PCC-FC vs regional CBF correlations were observed in bilateral PC, precuneus, VC, DLPFC, and mOFC, indicating a direct CBF modulation to the resting PCC-FC within the DMN (bilateral PA and precuneus), visual system, and the executive cognitive network (DLPFC and OFC). Similar to what was reported in [57], dACC-FC and vACC-FC (to save space, the latter was not displayed in Results) showed different networks. Only dACC-FC demonstrated reliable (across two time points) correlations to regional CBF which were located in bilateral PFC, left inferior and superior temporal cortex. Reproducible vACC-FC vs CBF correlations were identified in bilateral PFC, PCC, and superior temporal cortex when the significance level was reduced to p<0.05. This difference of regional ACC-FC vs regional CBF association might be induced by the small sample size involved in this study as well as the use of CASL, which is noisier than current methods [58]. No correlations were found between global CBF and global or regional FC, indicating no linear modulations of global CBF to FC. Since CBF modulations on regional FC were only observed in certain regions, the region constrained CBF vs FC correlations are likely suppressed when considering the whole brain average, which might explain why there were no correlations between global CBF and FC.

Stable correlations between regional CBF and SBA coherence (ReHo) or the low frequency fluctuation magnitudes (ALFF) were demonstrated in most of the brain cortex. The ReHo/CBF ratio and ALFF/CBF ratio-based analyses also suggest that a spatially uniform and stable linear relation between regional CBF and the two local SBA measures exists in GM, except for VC and precuneus. Global CBF showed no significant correlations to both regional ReHo, suggesting that regional SBA is not linearly modulated by the overall brain energy supply. A trend of correlation was found between the global CBF and global ReHo or global ALFF, which can be understood from the massive regional CBF vs ReHo or ALFF correlation as the correlation of global CBF vs global ReHo or ALFF can be approximated to certain extent by a summation of the regional CBF vs regional ReHo or ALFF. As compared to the regional FC vs regional CBF correlation, regional ReHo and ALFF showed spatially more distributed correlations to regional CBF. FC is derived from the correlation of any brain voxel's time series to that of the seed region, and might be modulated by CBF of the current voxel as well as that of the seed. Consequently, regional CBF might contribute only certain part of the FC variations across subject, which explains why regional FC vs regional CBF showed correlations in fewer brain regions than regional ReHo and ALFF vs regional CBF.

**Figure 10:**
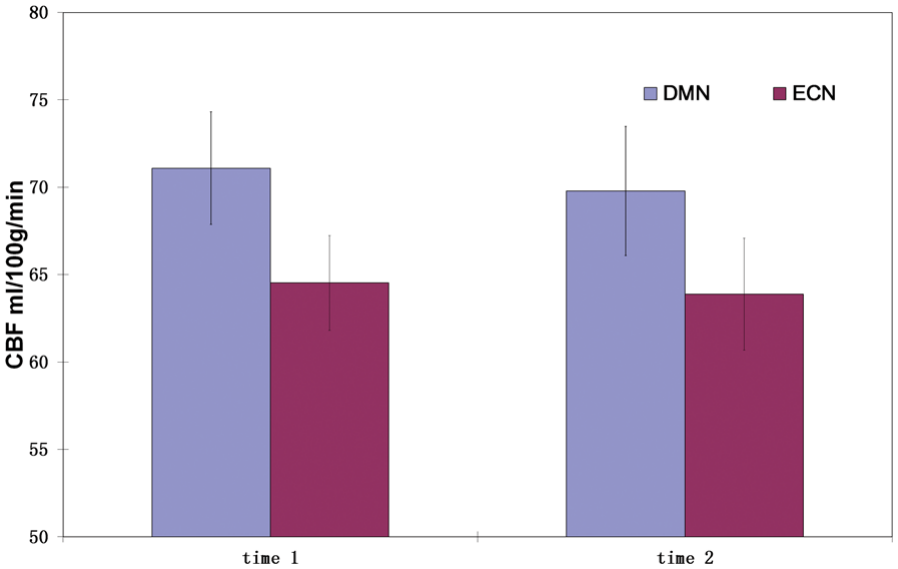
Default Mode Network (DMN) and executive control network (ECN) presenting significant (p<0.0004) different CBF at both sessions.

The value of these SBA vs CBF associations is twofold. First, it helps to link the apparent fMRI-derived parameters to a physiological meaningful measure. Though resting BOLD fMRI is assumed to be able to capture resting brain activity [2]–[12], these fMRI-derived parameters using either SRFC, ReHo, ALFF or even ICA cannot directly refer any physiological meanings. As baseline CBF reflects the baseline brain energy demand, relating those measures to CBF provides a way to appreciate their physiological underpinnings in brain metabolism. Second, it provides a way to normalize these purely data-dependent SBA metrics by using resting regional CBF, or alternatively the variations due to regional CBF can be removed in order to improve across subject comparisons, which might be very useful for clinical treatment or medicine study since both treatment and medicine can change regional CBF. Various brain networks might also be ranked or quantified using their CBF values. For example, in an additional analysis, we identified two resting brain networks, default mode network (DMN) and executive control network (ECN), using ICA on the BOLD data. We found that DMN regions showed higher mean CBF than ECN regions consistently at both time points (Fig. 10).

It is worth to note that the significant FC vs CBF correlation clusters appeared to be small, which raises a concern of noise interference. To reduce noise confounds, we used several preprocessing strategies including filtering, WM/CSF and global signal regression. More importantly, we used test-retest data from the same cohort of subjects with careful screening for any possible factors that may affect blood flow or even resting brain activity. As FC measures the correlations between two spatially distinct regions, it might be modulated by CBF from both regions.

Two limitations exist in this CBF-SBA association study. First, the data sample size is moderate and an uncorrected significance level was used for thresholding the results. Second, the retest data were acquired 2 months later. The small sample size might explain why we did not find any significant (even an uncorrected threshold was used) correlation between vACC FC and regional CBF and why we did not find any correlation between SBA and the global CBF if there were. The multiple comparison issue applies to the FC vs CBF correlation analysis but not to the ReHo vs CBF or ALFF vs CBF analyses since the suprathreshold clusters of the correlation of ReHo and ALFF to regional CBF survived the false detection rate (FDR)-based multiple comparison correction (q<0.05) [59], [60]. For FC vs CBF correlation analysis, although 15 subjects might not have enough power to reveal all possible SBA-CBF correlations, our findings were based on within-subject test-retest data, which partly compromised the sample size issue since these SBA-CBF associations repeated in 15 subjects should be reproducible when more subjects are recruited. A larger sample size and a more stringent thresholding criterion will be required in future work to confirm the findings reported here.

The test-retest data used in this paper were acquired in a project designed to test the stability of SBA measures and resting CBF with a long time interval (results were reported in a separate paper). Although a 2 month gap could conceivably introduce physiological or psychological variations to the data, we still found repeated SBA-CBF associations. One reason could be that these associations are stable over this duration in healthy subjects. Another explanation could be that the physiological and psychological variations affect SBA and CBF in the same way so their effects on the SBA-CBF correlation are canceled. Although we have screened for caffeine use and neurological disorders, several other factors, including breathing pattern, consumption of alcohol or caffeine, blood pressure, and activities of the autonomic nerve systems could alter blood flow. Since resting BOLD signals are modulated by CBF [61], [62], CBF fluctuations will likely be propagated into the resting BOLD-based SBA measures, resulting in apparent SBA-CBF correlations. One way to reduce these artificial SBA-CBF correlations is to use relative CBF maps instead of absolute CBF maps in the correlation analysis. Using the relative CBF maps (obtained by dividing absolute CBF map by the whole brain average absolute CBF) we found very similar correlation results (data not shown), which suggests that the demonstrated SBA-CBF correlations are not affected by those physiological variations and supports the findings of no-significant correlations of regional SBA to global CBF.

Various additional network-related measures [63]–[65] have also been used to assess SBA. Limited by the scope of this paper, we did not include them, though future work will cover the relations between more resting BOLD-based SBA measures like functional brain network properties [66]. Resting ASL MRI has been demonstrated to be capable for assessing SBA [42], [67]. But we did not include the ASL MRI-based SBA measures due to the limit of space.

In summary, we investigated the correlations between different SBA measures with CBF and their test-retest reproducibility. In our analysis, reliable correlations were demonstrated between CBF and PCC FC as well as dACC FC in many of the significant FC regions, respectively. Reliable correlations to regional CBF were found in ReHo and ALFF in most of gray matter area. No correlations were found between global CBF and these SBA measures. These results demonstrate that dynamic SBA measures based on BOLD fMRI are related to the static baseline CBF, which confirms a link between these apparent BOLD fMRI-based metrics and underlying SBA.

## Supporting Information

File S1 **The abstract presented in the 18th Annual Meeting of the Organization for Human Brain Mapping, June, 2012 in Beijing, China.**
(DOC)
