# Supplementary material for: Relations between BOLD fMRI-Derived Resting Brain Activity and Cerebral Blood Flow
Source: PLoS One. 2012 Sep 21;7(9):e44556. doi: 10.1371/journal.pone.0044556 (PMC3448607; doi:10.1371/journal.pone.0044556)
Supplement: File S1 — The abstract presented in the 18th Annual Meeting of the Organization for Human Brain Mapping, June, 2012 in Beijing, China. (DOC) [file pone.0044556.s001.doc]

**The Correlation between Functional Connectivity and Cerebral Blood Flow and its Reliability**

Zhengjun Li, BS ^1,2^, John Pluta, BS ^3^, Anna Rose Childress^2^, Ze Wang, PhD ^2,4*^

^1^ Department of Biomedical Engineering, Shanghai Jiao Tong University, China, People's Republic of, ^2^ Department of Psychiatry, University of Pennsylvania, USA, ^3^ Department of Neurology, University of Pennsylvania, USA, ^4^ Department of Bioengineering, School of Engineering and Applied Science, University of Pennsylvania, USA.

* correspondence should be addressed to:

Ze Wang, Ph. D,

Department of Psychiatry, School of Medicine, University of Pennsylvania,

3900 Chestnut Street, Philadelphia, PA 19104, USA,

Tel: 215-222-3200 ext 123 Fax: 215-386-6770

E-mail: [zewang@mail.med.upenn.edu](mailto:zewang@mail.med.upenn.edu)

Grant Support:

This work was supported by NIH grants: 1R03DA023496-01A1, DA015149, RR02305, and [4R33DA026114-03](http://projectreporter.nih.gov/project_info_details.cfm?aid=8087578&icde=5860093).

**Introduction**

The resting fMRI-based functional connectivity (FC) between different brain regions is assumed to reflect the spontaneous brain activity which accounts for most of the enormous energy consumptions in the brain . Cerebral blood flow (CBF) represents the major energy supply for the brain, and therefore provides a way to verify the association of resting FC to brain energy expenditure. In this work, we explored the association between resting FC and quantative CBF measured with arterial spin labeled (ASL) perfusion MRI and its reliability.

**Methods**

15 young healthy subjects (mean age = 25, range = 20-35, SD = 4.75, 8 male) were scanned in a 3-T Siemens whole-body scanner with signed written consent form approved by local IRB twice with 2 months apart. High-resolution structural images, blood-oxygen-level-dependent (BOLD) fMRI images and continuous ASL images were acquired consecutively while subjects were asked to lie still in the scanner at rest and keep eyes open.

BOLD data preprocessing was performed using SPM (http://www.fil.ion.ucl.ac.uk/spm) based batch scripts. The ASL mean CBF map was calculated using the ASL data processing toolbox, ASLtbx . Diffeomorphic Anatomical Registration Through Exponential Lie Algebra (DARTEL) was used to warp each individual’s BOLD and mean CBF images to the local template space generated based on all subjects’ structural images.

Seed regions of interest (ROI, sphere of 6mm radius) were defined in the posterior cingulate cortex (PCC) and anterior cingulate cortex (ACC, 3 in ventral ACC and 1 in dorsal ACC) using the Pickatlas utility. FC maps were calculated based on the mean signal of each ROI, and Group One sample T test was performed and thresholded at p < 0.001, uncorrected for multiple comparison.

For each intracranial voxel, CBF-FC correlations was calculated for each seed-FC and each scan session.

Regression analysis was also performed to check the relation between global CBF and regional FC for each session.

Correlation results were thresholded at r > 0.683 (p < 0.005, uncorrected for multiple comparison) and cluster size > 30 voxels.

**Results**

The group level PCC FC and ACC FCs (Fig. S1c and S1d, Fig. S2c and S2d) were similar to those repeatedly reported in the literatures .

CBF vs PCC-FC correlations were found in orbitofrontal cortex (OFC), visual cortex, PCC/precuneus, bilateral parietal cortex (PA), and left dorsal lateral prefrontal cortex (DLPFC) at both scan sessions (Fig. S1a and S1b). Most of these posoitive correlated voxels are in the significant PCC FC regions shown in Fig. S1c and S1d. Correlations in other brain regions were not repeated in the retest session.

No significant CBF correlations were found for vACC FCs using the 3 vACC ROIs. CBF vs dACC-FC correlations were consistently demonstrated in bilateral prefrontal cortex in both sessions (Fig. S2a and S2b). These regions were in the significant dACC FC regions as shown in Fig. S2c and S2d.

No significant correlation was found between global CBF and the FCs.

**Conclusions**

Reliable correlations were found between regional CBF and PCC FC and dACC FC in the significant FC areas, respectively, whereas no significant correlation was found between CBF and vACC FCs, suggesting different regional CBF modulations on the FC networks defined by different seed ROIs. The FC difference and CBF-FC correlation difference between vACC-FC and dACC-FC might reflect brain function difference in those regions (emotion/cognitive) . No significant correlations between global CBF and the FCs suggest that resting FC is unlikely to be modulated by whole brain CBF. Further investigations are required for CBF correlation with other FC networks and other measures of resting brain.

**Figures**

**
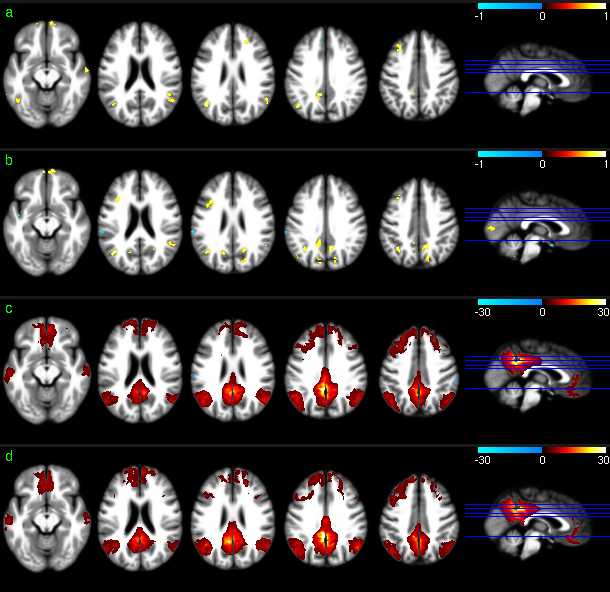
**

Fig S1. Cerebral Blood Flow (CBF) vs posterior cingulate cortex functional connectivity (PCC FC) correlation maps based on data acquired at a) session 1 and b) session 2 (r>0.683, p<0.005 uncorrected). Group One sample T test maps of PCC FC maps of c) session 1 and d) session 2 (p<0.001, uncorrected).


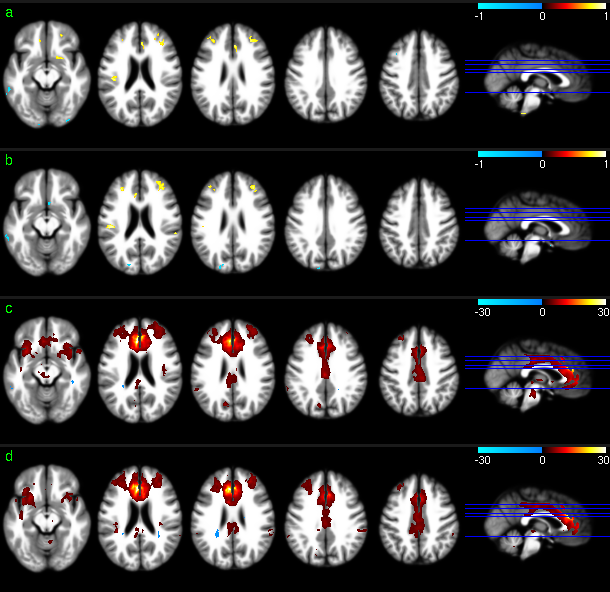


Fig S2. Cerebral Blood Flow (CBF) vs dorsal anterior cingulate cortex functional connectivity (dACC FC) correlation maps based on data acquired at a) session 1 and b) session 2 (r > 0.683, p < 0.005 uncorrected). Group One sample T test maps of dACC FC maps of c) session 1 and d) session 2 ( p< 0.001, uncorrected).

**Allman J, Hakeem A, Erwin J, Nimchinsky E, Hof P. (2001): The anterior cingulate cortex. The evolution of an interface between emotion and cognition. Annals of the New York Academy of Sciences 935:107-117.**

**Ashburner J. (2007): A fast diffeomorphic image registration algorithm. Neuroimage 38(1):95-113.**

**Biswal B, Yetkin FZ, Haughton VM, Hyde JS. (1995): Functional connectivity in the motor cortex of resting human brain using echo-planar MRI. Magn Reson Med 34(4):537-541.**

**Detre JA, Leigh JS, Williams DS, Koretsky AP. (1992): Perfusion imaging. Magn Reson Med 23(1):37-45.**

**Fox MD, Raichle ME. (2007): Spontaneous fluctuations in brain activity observed with functional magnetic resonance imaging. Nat Rev Neurosci 8(9):700-711.**

**Maldjian JA, Laurienti PJ, Kraft RA, Burdette JH. (2003): An automated method for neuroanatomic and cytoarchitectonic atlas-based interrogation of fMRI data sets. Neuroimage 19(3):1233-1239.**

**Margulies DS, Kelly AMC, Uddin LQ, Biswal BB, Castellanos FX, Milham MP. (2007): Mapping the functional connectivity of anterior cingulate cortex. Neuroimage 37(2):579-588.**

**Raichle ME, Mintun MA. (2006): Brain work and brain imaging. Annu Rev Neurosci 29:449-76.**

**Wang Z, Aguirre GK, Rao H, Wang J, Fernández-Seara MA, Childress AR, Detre JA. (2008): Empirical optimization of ASL data analysis using an ASL data processing toolbox: ASLtbx. Magn Reson Imaging 26(2):261-269.**
